# The Effects of Coronavirus Disease 2019 Outbreak on Medical Students

**DOI:** 10.3389/fpsyt.2021.637946

**Published:** 2021-03-16

**Authors:** Kadir Bilgi, Gamze Aytaş, Utku Karatoprak, Rümeyza Kazancıoǧlu, Semra Özçelik

**Affiliations:** Faculty of Medicine, Bezmialem Vakif University, Istanbul, Turkey

**Keywords:** medical students, COVID-19, anxiety, depression, education

## Abstract

**Introduction:** The coronavirus disease 2019 (Covid-19) pandemic has influenced the whole world, where after the first case was diagnosed in Turkey, educational activities were suspended and partial curfews were implemented. This study was conducted to assess the concerns faced by the medical students about their professional life due to the disrupted educational activities and related psychological effects.

**Methods:** This is a cross-sectional survey study, conducted with self-administered questionnaires on Bezmialem Vakif University medical students, during the pandemic. The questionnaire consists of queries about demographics, environmental factors, Generalized Anxiety Disorder-7 (GAD-7), and Patient Health Questionnaire-9 (PHQ-9) scales.

**Results:** A total of 178 students participated in the study, with a female-to-male ratio of 5:2. Of the total respondents, 19.7% were experiencing severe anxiety, 17.4% moderate anxiety, and 37.1% mild anxiety, according to the GAD-7; and 13.5% of the respondents were experiencing severe depression, 21.9% moderate–severe depression, and 23% mild depression according to the PHQ-9. There was no statistically significant difference between the grades in terms of GAD-7 or PHQ-9 scores. Male participants were more likely to have suicidal thoughts (*p* = 0.013). According to our study, the factors with the highest influence on students were as follows: “Major changes in personal life,” “Disruption in educational activities,” and “Covid-19 related anxiety of loss of relatives and contamination or infection.” On average, women voted higher points for “Covid-19 related anxiety of loss of relatives and contamination.”

**Conclusions:** We found that a significant portion of students regardless of their year in medical school were profoundly affected by the pandemic process as is shown by their anxiety and depression scores. The disruption in educational activities is one of the main factors of these effects, and we believe that these should not be ignored, as they could in the future lead to a series of problems for medical education and students alike.

## Introduction

In December 2019, several cases of pneumonia of unknown origin were first reported in Wuhan City, China. The agent responsible for these cases was later identified as a novel respiratory virus from the *Coronaviridae* family of viruses that was later named severe acute respiratory syndrome coronavirus 2 (SARS-CoV-2). Soon the influence of the novel virus was spreading throughout the whole world, as on 11 March 2020, the first case of coronavirus disease 2019 (Covid-19) was reported in Turkey (1), and on the same day, the World Health Organization (WHO) has declared the outbreak as a pandemic (2).

On 13 March 2020, several measures were implemented in Turkey including the suspension of educational and internship activities for 3 weeks at first and later extended for an undetermined period. Most public spaces were closed, and weekend curfews were implemented in the major population centers. After the initial 3 weeks, educational activities in medical schools have resumed in the form of online education; however, students could not engage in clinical education. According to the official website of the Ministry of Health of Turkey, there were 164,769 confirmed cases (including 934 severe cases) of Covid-19 in Turkey as of 1 June 2020 (1).

During this period, many studies were conducted around the world on the general population, which found that the prevalence of depressive symptoms ranged from 14.6 to 48.3% and the prevalence of anxiety symptoms ranged from 6.33 to 50.9% (3). Another study recruited health-care workers from major health-care institutions in five countries in the Asia-Pacific region. In these countries, among health-care workers, depression prevalence was 0.8–14.3% and anxiety prevalence was 0.8–6.8%. A study also highlights that the varied prevalence of psychological adversity among health-care workers is independent of the burden of Covid-19 cases within each country (4). There are also different studies in various populations such as psychiatric patients and general workforce (5, 6).

When considering the medical students, various mental health problems were studied and documented before the pandemic, and two meta-analyses on this subject have found the prevalence of anxiety to be 33.8% among medical students globally and a global depression prevalence of 28.0% among medical students (7, 8).

During the pandemic, authorities and institutions considered the possibility of involving medical students in the delivery of health care to support health-care system. Tran et al. proposed that to conduct fast and effective responses in the pandemic, universities should improve their training curriculums by incorporating field epidemiological practicum, as governments should develop policies and protocols that specify medical students' roles and responsibilities (9). Since the pandemic is ongoing, the approach used in Vietnam might be helpful for other resource-scarce settings in conducting active and prompt responses during the pandemic.

The uncertainty surrounding every aspect of this period, strict isolation measures, and the disruption of education are expected to influence the mental health of undergraduate students. Such effects have been demonstrated on medical students in previous studies during the SARS, Middle East respiratory syndrome (MERS), and H1N1 influenza outbreaks (10–13). These factors may also have an impact on the professional perceptions and behaviors of medical students.

Our aim in this study has been to quantitatively measure the mental health effects of the Covid-19 pandemic on medical students and to provide insights about the students' perception of the pandemic.

## Materials and Methods

This is a cross-sectional observational study, conducted with self-administered questionnaires on Bezmialem Vakif University Medical Faculty students, between 1 and 18 June 2020.

The questionnaire used in our study consisted of two main sections. The first part included demographical information including the contact, diagnosis, risk groups of Covid-19, and the participants' behavior during the pandemic until 1 June. The second part of the questionnaire consisted of the psychological effects of the pandemic and Generalized Anxiety Disorder-7 (GAD-7) and Patient Health Questionnaire-9 (PHQ-9) scales to quantitatively measure the mental health effects of the Covid-19 pandemic. The questions were all presented in Turkish to the participants, as validated translations were used for the GAD-7 and PHQ-9 scales (14, 15).

The GAD-7 includes seven items based on the seven core symptoms, and it inquires the frequency with which respondents suffered from these symptoms within the last 2 weeks (16). Respondents report their symptoms using a 4-item Likert rating scale ranging from 0 (not at all) to 3 (almost every day), such that the total score ranges from 0 to 21. The GAD-7 is a well-validated screening instrument, and it has demonstrated excellent internal consistency (Cronbach's α = 0.911) (17).

The PHQ-9 is a nine-item depression scale based on the depression symptoms, and it inquires the frequency with which respondents suffered from these symptoms within the last 2 weeks (18). As a severity measure, the PHQ-9 score can range from 0 to 27, since each of the nine items is scored from 0 (not at all) to 3 (nearly every day). PHQ-9 has been shown to be a reliable measure of depression (19).

The questionnaire was sent to the participants *via* Google Forms application over the Internet, and their responses were collected in the same measure.

The SPSS 21.0 software package (SPSS, Inc., Chicago, IL, USA) was used to perform statistical analyses. The distribution of the data was examined by the Shapiro–Wilk test. Normally distributed data are presented as the mean ± standard deviation, non-normally distributed data are presented as the median with interquartile range, and categorical variables are presented as frequency and percentages. Comparisons between the groups were analyzed by the Mann–Whitney *U*-test, Kruskal–Wallis *H*-test, Friedman test for non-normally distributed variables, and the Pearson chi-square test for categorical variables. Correlations between variables were analyzed by the Spearman correlation test for non-normally distributed variables. All statistical analyses were performed and reported at the α = 0.05 significance level, and all *p*-values were two-tailed.

## Results

A total of 178 students participated in the study. Participants' distribution by grade is presented in Table 1. The female (*n* = 127) to male (*n* = 51) ratio of the participants was calculated as 5:2. Since the questionnaire was directly sent to all students of the medical faculty, the difference between genders is a result of students' willingness to participate as shown in the literature (20). The median age of the participants was calculated as 21 [20–23] years, where the youngest participant was 18 and the eldest 25.

**Table 1:** Demographics.

**Grade**	**1st year**	**2nd year**	**3rd year**	**4th year**	**5th year**	**6th year**
	23 (12.9%)	39 (21.9%)	27 (15.2%)	24 (13.5%)	41 (23.0%)	24 (13.5%)
Accommodation	I am staying alone	I am staying with my family		I live in the dorms	I am staying in a student house	
	4 (2.2%)	169 (94.9%)		1 (0.6%)	4 (2.2%)	
Covid-19 contact	I had close contact with someone I knew that has Covid-19		I had close contact with someone I suspect that has Covid-19		I did not have close contact	
	2 (1.1%)		10 (5.6%)		166 (93.3%)	
Covid-19 diagnosis	I was diagnosed with Covid-19		One of my relatives received the Covid-19 diagnosis		None of the above	
	5 (2.8%)		22 (12.4%)		151 (84.8%)	
How many times a week did you leave the house? (until June 1)	I never go out	I go out once	I go out twice	I go out 3 times	I go out 4 times or more	
	55 (30.9%)	71 (39.9%)	30 (16.9%)	14 (7.9%)	8 (4.5%)	

*Covid-19, coronavirus disease 2019*.

Participants were asked a series of demographic questions; the answers are presented at length in Table 1. In terms of Covid-19 risk groups, while nine (5.1%) of the participants stated that they were in one of the risky groups themselves, the number of people who stated that at least one member of their family or the people they live within a high-risk group was 117 (65.7%). In terms of accommodation, the overwhelming majority (169 participants, 94.9%) stated that they lived with their family during the pandemic, where only four participants (2.2%) reported that they were living alone. On the Covid-19 contact question, 12 participants (6.7%) stated that they had close contact with a suspected or diagnosed Covid-19 patient, while 22 (12.4%) of the participants stated that one of their relatives received a Covid-19 diagnosis, and five (2.8%) participants stated that they were diagnosed with Covid-19 themselves. Fifty-five (30.9%) of the participants stated that they chose not to leave their house until 1 June.

To assess the perceptions of the students, we asked a series of Likert-type questions, where the answers are presented in Table 2 at length. One hundred two (57.30%) participants agreed or strongly agreed with the statement “I think that I was adequately informed about infectious diseases and prevention methods during my medical education.”

**Table 2:** General Likert questions.

**Questions**	**I strongly disagree**	**I disagree**	**Undecided**	**I agree**	**I strongly agree**
I believe it is my responsibility to inform the people around me about Covid-19	4 (2.2%)	9 (5.0%)	24 (13.4%)	78 (43.8%)	63 (35.3%)
I thought of leaving my medical school education during the Covid-19 pandemic	104 (58.4%)	49 (27.5%)	9 (5.0%)	8 (4.4%)	8 (4.4%)
I am worried that I will not be able to complete my internships on time	19 (10.6%)	26 (14.6%)	39 (21.9%)	44 (24.7%)	50 (28.0%)
I have difficulty starting new scientific projects	11 (6.1%)	25 (14.0%)	45 (25.2%)	46 (25.8%)	51 (28.6%)
I have difficulty maintaining my old projects	12 (6.7%)	27 (15.1%)	48 (26.9%)	39 (21.9%)	52 (29.2%)
I think I will be more comfortable when requesting a letter of recommendation	36 (20.2%)	29 (16.2%)	96 (53.9%)	10 (5.6%)	7 (3.9%)
I think that I was adequately informed about infectious diseases and prevention methods during my medical education	12 (6.7%)	26 (14.6%)	38 (21.3%)	72 (40.4%)	30 (16.8%)
Covid-19 pandemic caused my priorities to change in terms of specialty choice	46 (25.8%)	54 (30.3%)	45 (25.2%)	19 (10.6%)	14 (7.8%)
I think the epidemic process will negatively affect my success in *TUS*	40 (22.4%)	40 (22.4%)	40 (22.4%)	30 (16.8%)	28 (15.7%)
Covid-19 pandemic made me look at field of medicine more positively	13 (7.3%)	17 (9.5%)	56 (31.4%)	47 (26.4%)	45 (25.2%)

*Covid-19, coronavirus disease 2019. T.U.S., Tipta Uzmanlik Sinavi (in Turkish; English-Medical Specialty Examination)*.

On the other hand, 141 (79.2%) of the participants agreed or strongly agreed with the statement “I believe it is my responsibility to inform the people around me about Covid-19.” However, only 84 (59.5%) of them stated that they were adequately informed about infectious diseases in the question mentioned above.

One hundred fifty-three (85.9%) participants disagreed or strongly disagreed with the statement “I thought of leaving my medical school education during the Covid-19 pandemic,” 89 (50.0%) participants agreed or strongly agreed with the statement “Covid-19 pandemic made me look at the field of medicine more positively,” and only 56 (31.5%) participants selected the neutral option.

One hundred (56.17%) participants disagreed or strongly disagreed with the statement “Covid-19 pandemic caused my priorities to change in terms of specialty choice,” and those in a risk group for Covid-19 have agreed with this statement significantly more than those who were not (*p* < 0.001). The majority of the participants agreed or strongly agreed with the statements “I have difficulty starting new scientific projects” and “I have difficulty maintaining my old projects,” 97 (54.4%) and 91 (51.1%) participants, respectively, and these participants had significantly higher GAD-7 and PHQ-9 scores.

The 1st-year students rated statistically significantly higher on the Likert scale question “Covid-19 pandemic made me look at the field of medicine more positively” than the sixth-year students (*p* = 0.012).

One hundred thirty-seven (77%) of the participants stated that they had no suicidal thoughts within the last 14 days. In accordance with the literature, male participants were more likely to have suicidal thoughts (21) (*p* = 0.013).

**Figure 1:**
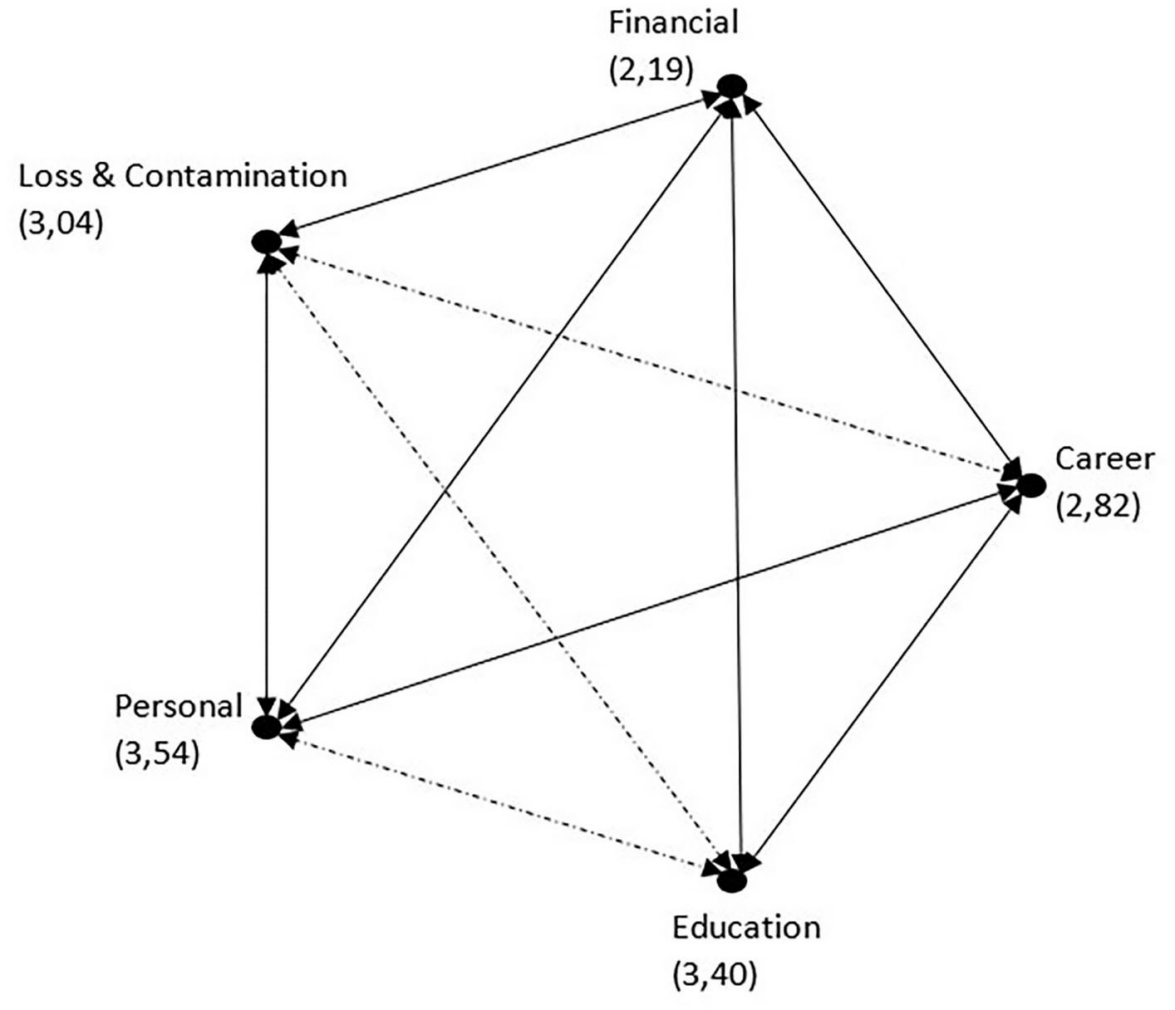
Likert questions' pairwise comparisons. Each node shows the sample average rank. Significant differences are shown with continuous lines.

We asked participants to specify how much they were affected psychologically by the five major factors on a Likert scale. A Friedman's test showed that there was a significant difference between these factors (*p* < 0.001). According to the test, the mean ranks of the factors were 3.54 for “Major changes in personal life,” 3.40 for “Disruption in educational activities,” and 3.04 for “COVID-19-related anxiety of loss of relatives and contamination or infection.” *Post-hoc* tests were performed and showed that the “Major changes in personal life” is rated significantly higher than the rest of the factors except “Disruption in educational activities” (*p* < 0.005 for all). The rest of the pairwise comparisons are presented in Figure 1. On average, women voted higher points for “COVID-19-related anxiety of loss of relatives and contamination,” which was in accordance with the present literature (22). All five Likert questions had significant correlations with the GAD-7 and PHQ-9 scores (for all; *r* > 0.250, *p* < 0.001) (Table 3).

**Table 3:** Likert questions' correlations with the scales.

		**GAD-7 scores**	**PHQ-9 scores**
Disruption in educational activities	Correlation coefficient	0.480	0.457
	Sig. (2-tailed)	<0.001	<0.001
Concerns about career plan (domestic/foreign specialty training etc.)	Correlation coefficient	0.331	0.378
	Sig. (2-tailed)	<0.001	<0.001
Covid-19-related anxiety of loss of relatives and contamination or infection	Correlation coefficient	0.347	0.259
	Sig. (2-tailed)	<0.001	<0.001
Financial concerns	Correlation coefficient	0.440	0.442
	Sig. (2-tailed)	<0.001	<0.001
Major changes in personal life	Correlation coefficient	0.534	0.391
	Sig. (2-tailed)	<0.001	<0.001

*All statistical analyses in this table were performed with the Spearman correlation test*.

*GAD-7, Generalized Anxiety Disorder-7; PHQ-9, Patient Health Questionnaire-9; Covid-19, coronavirus disease 2019*.

The median score of the GAD-7 scale was 7 [4–13], and the median score of the PHQ-9 scale was 11 [7–17] for the whole study group. According to the GAD-7 scale, 35 (19.7%) participants had severe anxiety, 31 (17.4%) participants had moderate anxiety, and 66 (37.1%) participants had mild anxiety. According to the PHQ-9 scale, 24 (13.5%) participants had severe depression, 39 (21.9%) participants had moderately severe depression, and 41 (23.0%) participants had moderate depression

There was no statistically significant relationship between the age, genders, and grades in terms of GAD-7 or PHQ-9 scores. The GAD-7 scores of participants with at least one individual who is in a high-risk group in their family were statistically significantly higher (*p* = 0.047). There was no statistically significant difference between the participants' GAD-7 and PHQ-9 scores in terms of how frequently they went out, what their Covid-19 diagnosis status is, or their Covid-19 contact status; but the contact group was too small for a healthy analysis. Both PHQ-9 and GAD-7 scores had positive correlations with the Likert questions “I am worried that I will not have a chance to complete my internships on time” (*r* = 0.220, *p* = 0.003/*r* = 0.221, *p* = 0.003) and “I think the epidemic process will negatively affect my success in *T.U.S*.” (*r* = 0.178, *p* = 0.017/*r* = 0.298, *p* < 0.001) (*T.U.S*. is the examination for residency training in Turkey).

## Discussion

Our aim in this study was to demonstrate the mental health effects of the Covid-19 pandemic on medical students. As we have shown in the results, 66 (37.1%) participants have scored over 10 in the GAD-7 scale, which is interpreted as severe or moderate anxiety, and 63 (35.4%) participants have scored over 15 in the PHQ-9 scale, which is interpreted as severe or moderately severe depression. Recently, two new studies on medical students have found out that 46.1–56.1% of medical students scored 10 or more in the GAD-7 scale, thereby identified as having moderate or severe symptoms of anxiety, and 52.5–64.4% of them scored 10 or more in the PHQ-9 scale, thereby identified as having moderate or severe symptoms of depression (23, 24). One study from China on the other hand found that 7.4% of medical students scored 10 or more in the GAD-7 scale and that 11.1% of them scored 10 or more in the PHQ-9 scale (25). From these comparisons, we can infer that our study population, although having lower rates of depression and anxiety than the other medical students around the globe, did not significantly differ from them and that these are medical students' problems in general.

Through the results of this research, we can conclude that some factors have a relationship with the PHQ-9 and GAD-7 scores either positively or negatively. We have demonstrated that being in a high-risk group had a positive relationship with PHQ-9 scores, as having a relative who belongs to such group had a positive relationship with GAD-7 scores. We have seen that worries about not being able to complete their internships, effects of the pandemic on the *T.U.S*. success, difficulty of starting a new scientific project, and maintaining the old ones had positive relationships for both scale scores. Contrary to some of the present literature, we found no significant difference between genders in terms of both scale scores (23).

We have demonstrated that participants self-identified “Major changes in personal life” and “Disruption in educational activities” as the most effective factors on their psychological well-being, the reliability of which is supported by these questions' strong correlations with both of the scale scores (Table 4).

**Table 4:** PHQ-9 and GAD-7 results.

**PHQ-9 score groups**	**Moderate depression** **(10–15)**	**Moderately severe depression** **(15–20)**	**Severe depression** **(>20)**
Participants	41 (23.0%)	39 (21.9%)	24 (13.5%)
**GAD-7 score groups**	**Mild anxiety** **(5 to 10)**	**Moderate anxiety** **(10 to 15)**	**Severe anxiety** **(>15)**
Participants	66 (37.1%)	31 (17.4%)	35 (19.7%)

*PHQ-9, Patient Health Questionnaire-9; GAD-7, Generalized Anxiety Disorder-7*.

As we mentioned in the *Results* section, most of the students have a more positive outlook on the field of medicine after the pandemic's effects, as most do not think their priorities have changed regarding specialty choice, and very few students thought of leaving the medical school. As a result of these, authors argue that the health risks and other effects of the Covid-19 pandemic did not affect the views of students about the medical field negatively, although the authors acknowledge that these are their personal interpretation and there could be other factors not considered here that require further inquiries to be explained.

We believe these health problems mentioned above need to be assessed and addressed by the institutions. There are various strategies that institutions have used before the pandemic to help medical students with anxiety and depression, but such measures may not be implementable in the time of a pandemic. There has already been some discussion on what can be done in terms of mental health strategies under these conditions (26) including digital cognitive behavioral therapy, which has been shown to be effective on other mental health problems and also has cost-effective solutions for its implementation (27, 28).

Ultimately, this study has some limitations of its own that need to be addressed. First, this was a cross-sectional study; thus, there were no data of the study group before the pandemic. Second, there was not enough participation to conduct a healthy statistical analysis in some groups for some comparisons such as students who are in the high-risk group and students who do not live with their families. More so, this study was conducted in just one center and may not represent the general condition of the medical students elsewhere in some respects. Finally, this study used a self-reported questionnaire to measure psychiatric symptoms and did not make any clinical diagnosis. The gold standard for establishing psychiatric diagnosis involves structured clinical interview and functional neuroimaging (29).

## Conclusions

We would like to conclude this article by stating that the psychological effects of the pandemic on medical students are quite significant, and the disruption in educational activities is one of the main factors of these effects, and we believe these should not be ignored as it could lead in the future to a series of problems for the medical education and students alike.

## Data Availability Statement

The raw data supporting the conclusions of this article will be made available by the authors, without undue reservation.

## Ethics Statement

The ethics approval was provided by Bezmialem Vakif University Non-Interventional Research Ethics Committee (No: 18/358). Participants were informed at the start of the questionnaire about that their consent would be assumed if they filled the questionnaire.

## Author Contributions

KB, GA, and UK have designed the study, acquired, analyzed, interpreted the data, drafted the work, and revised the manuscript. RK made contributions to the conception of the study, substantively revised the manuscript, and supervised the project. SÖ contributed to the manuscript and revised it. All authors read and approved the final manuscript.

## Conflict of Interest

The authors declare that the research was conducted in the absence of any commercial or financial relationships that could be construed as a potential conflict of interest.

## Abbreviations

GAD-7: Generalized Anxiety Disorder-7
PHQ-9: Patient Health Questionnaire-9
*T.U.S*.: Tıpta Uzmanlık Sinavı (in Turkish; *English-Medical Specialty Examination*)
WHO: World Health Organization.
